# Prophage induction and acetate availability are associated with distinct *Lactiplantibacillus plantarum* electrode responses

**DOI:** 10.1128/msystems.00183-26

**Published:** 2026-04-30

**Authors:** Aaron Leininger, Emily Mayo, Yuqing Yan, Wenyu Gu, Harold D. May, Zhiyong Jason Ren

**Affiliations:** 1 Department of Civil and Environmental Engineering, Princeton University 6740, Princeton, New Jersey, USA; 2 Andlinger Center for Energy and the Environment, Princeton University 6740, Princeton, New Jersey, USA; 3 Institute of Environmental Engineering, École Polytechnique Fédérale de Lausanne 27218, Lausanne, Switzerland; Politecnico di Torino, Turin, Italy

**Keywords:** fermentation, lactic acid bacteria, extracellular electron transfer, prophage

## Abstract

Lactic acid bacteria can use an electron transport system to pass reducing equivalents to extracellular mediators. This redox flexibility decouples carbon and electron balances and presents the opportunity to use electrodes to engineer fermentations. We show that growth with an anode may be a two-edged sword for the fitness of the model lactic acid bacterium *Lactiplantibacillus plantarum* grown in a chemically defined medium supplemented with quinones, depending on substrate and cultivation regime. In batch culture on glucose, the anode slows growth despite yielding an over four-fold higher ratio of acetate to lactate than the open-circuit control, a fermentation profile associated with higher ATP yield. In batch culture on the more reduced substrate mannitol, the anode facilitates growth by enabling the dissipation of reducing equivalents. However, when acetate is also present as an alternate electron sink, the anode prolongs the lag phase while maintaining a growth rate similar to the open-circuit control, despite the expected ATP cost of acetate-to-ethanol reduction. Under semi-continuous cultivation on mannitol in the absence of acetate, anode polarization reduces growth yield relative to open-circuit conditions. Although the anode promotes more energetically favorable fermentation patterns during batch growth on both glucose and mannitol, these are likely counterbalanced by prophage induction to affect population growth, as confirmed by transcriptomic analysis, extracellular DNA measurement, and transmission electron microscopy. Together, these results indicate that extracellular electron transfer associated with an anode is not necessarily beneficial for fermenters and prompt further inquiry into the context-dependent nature of this style of metabolism.

**IMPORTANCE:**

A hybrid metabolism has been described in widespread fermenters where energy conservation through substrate level phosphorylation is coupled with electron transfer to external electron acceptors via extracellular redox mediators. This mechanism shapes interactions in microbial systems and presents opportunities in biotechnology. The variable physiological effects observed here of using an electrode as an electron sink highlight both the potential and challenges of applying electrodes to regulate fermentations. We show that polarization of an anode can hinder *Lactiplantibacillus plantarum* growth and is associated with induction of prophages, increasing the rate of lysis and thereby possibly counteracting the benefits of metabolic flexibility and more energetic fermentation patterns. These results make a new connection between electron balancing and a mobile genetic element and suggest a dynamic, context-dependent role of these mediators as public goods.

## INTRODUCTION

Understanding and developing new controls for lactic acid bacteria (LAB) is a topic with relevance to biorefinery concepts, agriculture, food science, and animal and human microbiomes. There is increasing interest in incorporating cheap, renewable electrons into CO_2_ reduction, and coupling short-chain electrolysis products with bioprocesses like lactate-based chain elongation can produce petrochemical-replacing platform chemicals (1–4). LAB produce a wide variety of fermented foods and plays a key role in silage fermentation (5). In 2020, they fermented 138 million tons of corn silage in the United States (6). In human health, LAB are extant throughout the alimentary canal and engage in complex interactions with epithelial cells and other bacterial community members (7). Their ability to perform extracellular electron transfer (EET) is likely important to their interactions with other cells and the surrounding environment and presents opportunities for technological development.

The fermentation products of LAB depend on the substrate, environment, and growth stage and reflect the need for energy generation and redox balance. Homolactic bacteria predominantly produce lactic acid from hexose sugars via pyruvate and the Embden-Meyerhof-Parnas pathway, where the NADH produced from glycolysis is balanced by NAD^+^ from the reduction of pyruvate to lactate. Many LAB, such as the model fermenter *Lactiplantibacillus plantarum*, perform such homolactic fermentation on hexoses but exhibit metabolic flexibility in variable conditions. Fermentation of a reduced substrate like mannitol or sorbitol is not homolactic and requires production of a more reduced product like ethanol or alternative redox-balancing pathway because pyruvate reduction to lactate cannot regenerate sufficient NAD^+^ to sustain mannitol consumption.

Beyond pyruvate reduction to lactate, LAB regenerate NAD^+^ by producing other reduced metabolites, including reducing glucose to mannitol (8) and reducing environmental acetate to ethanol (9, 10). Acetate uptake and reduction can support growth on reduced substrates under air-exposed conditions, where the inactivation of pyruvate-formate-lyase may impair pyruvate-to-ethanol conversion (10, 11). LAB do not encode full respiratory pathways but can use oxygen via oxidases or uptake environmental heme to gain order-of-magnitude improvements in energy yield from substrate by enabling oxidative phosphorylation, as in *Streptococcus pyogenes* in the familiar case of streptococcal throat infections (12). These metabolic versatilities help balance carbon flux and redox requirements and confer competitive advantages under variable conditions.

The redox balance between substrate and products can be broken by participating in EET. LAB have long been known to engage in outward EET by using quinones (13, 14) and flavins (15). A flavin-based EET (FLEET) system present in diverse Gram-positive bacteria genomes was described in 2018 (16) and shown to increase the fermentation and growth of FLEET-encoding LAB through an energy conservation pathway that blends features of fermentation and respiration: generating ATP through substrate-level phosphorylation while also using a terminal electron acceptor such as an anode, via extracellular flavins, to regenerate NAD^+^ (17). More recently, *L. plantarum* was also shown to perform endogenous inward EET as a metabolic switch enabling energy conservation through denitrification after substrate depletion (18).

FLEET-style metabolism in *L. plantarum* requires a flavin as an extracellular shuttle and quinone to bridge the periplasmic space, both of which the organism is auxotrophic for. Flavins may be available from plant material (e.g., ca. 0.2 mg riboflavin per 100 g of spinach) (19) or from other microbes, such as *Shewanella* spp., that secrete flavins to facilitate EET (20, 21). Quinone autotrophies are common in organisms with obligate and conditional requirements, and their interspecies exchange facilitates functions including and beyond electron exchange (22). Quinone secretion is described in other LAB (14, 23) and in lactate secondary-fermenters like *Propionibacterium* spp. (24–26) and may be affected by redox conditions (24). *L. plantarum* can assimilate and modify these quinones to carry electrons between inner and outer proteins, enabling flavins to serve as extracellular shuttles, or use them as extracellular shuttles themselves (27), with EET activity logarithmically scaling across quinone concentrations ranging from 1 to 100mg L^−1^ (23). Quinones have three redox states, including a semiquinone radical intermediate associated with generation of reactive oxygen species and DNA damage.

Prophage elements, a major class of mobile genetic elements, are widely distributed in *L. plantarum* genomes (28), and some strains harbor intact phages that are inducible by oxidative but not acidic stress (29). To our best knowledge, phage induction rates in *L. plantarum* have not been quantified under growth conditions. *L. plantarum* WCFS1 prophages were identified in 2003 but were not released spontaneously in De Man-Rogosa-Sharpe (MRS) broth or by mitomycin C treatment (30). *Lactococcus lactis* has observed 0.08 to 1.76% spontaneous prophage induction rate under various stressors including nutrient availability, osmolarity, temperature, and acidity (31). These findings highlight prophages as dynamic mobile genetic elements whose induction frequencies likely influence population-level physiology and stress responses in *L. plantarum*.

Here, we examine the metabolic, transcriptomic, and physiological responses of *L. plantarum*, a common silage and food fermentation inoculant, to an anode in the presence of quinone. We address a central question for applied fermentative electroactivity: under which conditions does the anode help or hurt their growth? We hypothesized that (i) anodic electron transfer relieves redox constraints and affects growth depending on substrate oxidation state; (ii) alternative soluble electron acceptors, such as acetate, modulate the physiological response to the anode; and (iii) cultivation regimes influence the role of EET in population growth.

Accordingly, glucose and mannitol, two common substrates with different average carbon oxidation states, were examined under open-circuit (OC) and polarized anode conditions and under batch vs. semi-continuous cultivation. Furthermore, the role of acetate, an abundant metabolite in fermentation environments, was examined as an alternative electron acceptor that drives a different physiological response by *L. plantarum* to the electrode. These results improve understanding of the relevance of EET to fermentation and inform ecological relationships involving quinone sharing.

## MATERIALS AND METHODS

### Bioelectrochemical reactors

Experiments were conducted in 300-mL H-cell reactors with a 4- × 4- × 0.6-cm carbon felt working electrode attached with Ti wire (ɸ = 0.635 mm, McMaster-Carr, IL, USA). Electrode chambers were separated by cation exchange membrane (CMI-7000, Membranes International, Inc.), and the counter chamber contained a 304 stainless steel wire counter electrode (ɸ = 2.03 mm, McMaster-Carr). Reactors with working/counter electrodes were autoclaved with deionized water, drained, and fitted with 70% ethanol-washed Ag/AgCl (1 M KCl) reference electrodes (CHI111, CH Instruments, USA) before filling with filter-sterilized media (32). Anodic polarization was conducted with a multi-channel potentiostat (BioLogic VMP-3000, France) at +200 mV vs Ag/AgCl to provide a terminal electron acceptor for redox mediators. Open-circuit potentials (OCPs) were obtained manually during liquid sampling with a digital multimeter. A video tutorial of a similar bioelectrochemical experiment is available in a recent JoVE publication (33).

### Strains, media, and culture conditions

*Lactiplantibacillus plantarum* NCIMB8826 (ATCC, USA) from glycerol stocks was grown for 18–36 h in glucose MRS media (34) (Oxoid, UK) at 37°C with air headspace and mild shaking. Cells were harvested by centrifugation (5,200 *× g*/12 min/4°C) and washed twice with 1× Dulbecco’s PBS (MP Biomedicals, CA, USA).

All experiments used a filter-sterilized, MOPS-buffered chemically defined medium (CDM) (17) with Wolfe’s vitamins and minerals with either mannitol (mCDM; 22.772 g L^−1^) or glucose (gCDM; 10 g L^−1^) as substrate. 1,4-dihydroxy-2-naphthoic acid (DHNA) was added as a quinone source at 20 mg L^−1^. Acetate (500 mg L^−1^ / 8.47 mM) was added in some experiments as its sodium salt. Riboflavin was routinely added at 1 mg L^−1^ in Wolfe’s vitamins, or, where indicated, to approximately saturation (estimated at 85 mg L^−1^). The medium was not sparged or otherwise degassed in any experiments.

### Operation and sampling

H-cell reactors were operated with magnetic stirring at ambient temperature. Except in a semi-continuous culture experiment, working electrode chamber headspaces were continuously flushed with N_2_ at around 200 cm^3^/min, maintaining dissolved oxygen below 15 µg L^−1^ (Fig. S1). pH was manually adjusted to ~pH 6.5 following liquid analyte sampling by injecting 50–250 µL of 50% (vol/vol) NaOH in all experiments, unless otherwise indicated. In semi-continuous culture experiments, 25, 50, or 75% of the 300-mL medium volume was removed and replaced with fresh mCDM by sterile syringe at 24-h intervals.

### Chemical analyses

One milliliter samples were collected with sterile syringes and analyzed for optical density at 600 nm (OD_600_; Genesys 10S UV-Vis), pH (Orion 8220BNWI), and filtered through 0.22-µm filters prior to high-performance liquid chromatography (HPLC) analysis. Organic acids and alcohols were analyzed on an Agilent 1260 Infinity II HPLC with refractive index detector operated at 0.6 mL min^−1^ with 4 mM sulfuric acid as the eluent. Standards included glucose, mannitol, formate, acetate, lactate, ethanol, propionate, butyrate/isobutyrate, and valerate/isovalerate. No gaseous products were measured due to the continuous headspace purging. Dissolved oxygen was measured using PreSens optical sensor spots (SP-PSt3-YAU, Regensburg, Germany). Volatile solids of attached growth on electrodes were determined by overnight drying at 104°C and subsequent mass loss on ignition at 550°C. Extracellular DNA in filtered medium samples was quantified via fluorescence (excitation/emission 504/523 nm) using 5 µM SYTOX Green with calf thymus DNA standards.

### Transcriptome analyses

Exponential-phase transcriptomes of triplicate batch fermentations of pH-controlled CDM (acetate+) with saturation riboflavin were compared between anodically regulated and OC mCDM, and anodically regulated and OC gCDM (i.e., two pair-wise comparisons from a total of three conditions). Samples were harvested by centrifugation (10,000 *× g*/3 min/4°C) after crossing an OD of 0.20, and the resulting cell pellet was stored in 5 mL RNALater (Invitrogen, USA) at −20°C prior to extraction. Cell pellets were lysed, and total RNA extracted with the RNA Power Biofilm kit (Qiagen, Germany) with on-column DNase treatment. RNA quality was determined with an RNA Pico Kit on Agilent 2100 Bioanalyzer; all samples had high RNA integrity (RIN > 8) and were used for downstream analysis. After rRNA depletion, strand-specific cDNA libraries were prepared with the Apollo 324 automated library preparation system and sequenced for 138 cycles in a 100-nt Flowcell v1.5 on Illumina NovaSeq SP. Bioanalyzer analysis, rRNA depletion, library preparation, and sequencing were performed at the Princeton University Genomics Core Facility.

RNAseq read quality was examined with FastQC in Galaxy (35). Low-quality reads were trimmed by Trimmomatic (36) with a sliding window requiring an average quality score of 20 across four bases. Remaining reads were aligned to a concatenated file of *L. plantarum* NCIMB8826 genome and plasmids using Bowtie2 (37) (--sensitive). Next, featureCounts (38) was used to quantify reads aligned with annotated genes. Chimeric fragments were excluded, and only fragments with both reads aligned were allowed. DESeq2 (39) with positive count size factor estimation due to the sparsity of some genes [estimateSizeFactors(dds, type = “poscounts”)] was used to identify differentially expressed genes in R, using a Benjamini-Hochberg false discovery rate adjusted *P*-value of 0.05 and |log_2_ fold change| ≥1 as thresholds. Mean-variance analysis was conducted to check for systemic biases (Fig. S2). Differentially expressed genes (DEGs) are reported in Tables S1 and S2.

Prophage regions of *L. plantarum* NCIMB8826 were analyzed for completeness using PHASTEST Docker and the PHASTEST database (version Feb 24, 2023) (40). Gene set enrichment analysis from anodically-regulated vs OC fermentations of mannitol was performed using the fgsea R package (v1.30.0) on the two prophage regions, LP_RS02750-02935 and LP_RS10245-10375. Genes were ranked by the DESeq2 test statistic (stat), and set enrichment was reported as normalized enrichment score (NES) and Benjamini-Hochberg adjusted *P*-value.

### Electrochemical analyses

Charge equivalents of anode reduction were calculated by integrating chronoamperometric responses. Charge equivalents associated with acetate reduction were calculated from acetate consumption, the electron equivalent of acetate to ethanol reduction (*n* = 4 electrons), and the Faraday constant (96,485.3 A * s mol^−1^ electrons), as detailed in Appendix SA. All potentials are reported relative to the Ag/AgCl reference.

### Transmission electron microscopy

Media (gCDM [saturation riboflavin] and gCDM) at OD of 0.8 from anodically-poised reactors were centrifuged (1,350 *× g*/10 min/4°C). The pellet was suspended in 5-mL SM buffer with light vortexing. Then, 15 µL of sample was loaded to a 400-mesh carbon-covered copper TEM grid (Electron Microscopy Sciences) and stained with 2% uranyl acetate for 30 s. The specimen was imaged at 120 kV on a Talos L120C G2 transmission electron microscope. Phage particles were not readily detected in concentrates prepared with polyethylene glycol precipitation (29) or 100-kDa Amicon filters (41).

## RESULTS

### Anode polarization slows growth, changes fermentation products on glucose

**Fig 1:**
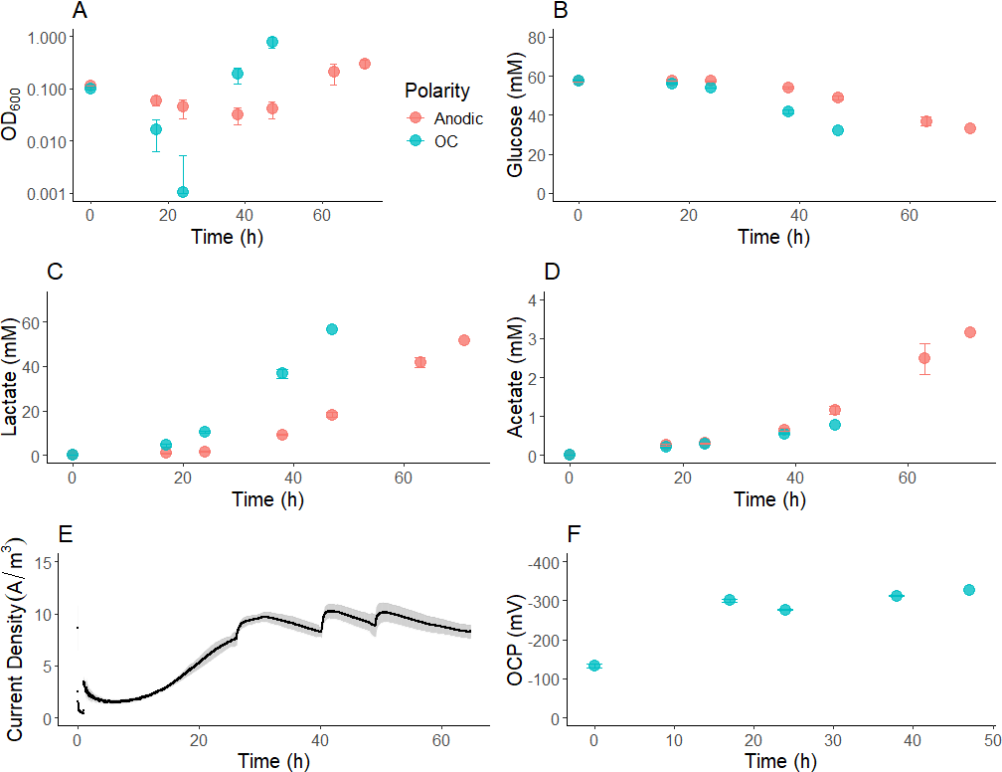
Anodic polarization impedes *L. plantarum* glucose fermentation in batch cultures. Fermentations were conducted in gCDM until OD_600_ ≥ 0.20. (**A**) OD_600_, (**B**) glucose, (**C**) lactate, (**D**) acetate concentrations, (**E**) chronoamperometric response, and (**F**) OCPs were measured from biological triplicates. Bars indicate the average ± standard deviation of triplicates. Panel A—lower bounds were truncated at OD_600_ = 10^−^³ for log scaling.

We evaluated the effect of anode polarization during batch fermentation of glucose. Anode polarization slowed the planktonic growth rate (Fig. 1A), glucose utilization rate (Fig. 1B), and lactate production rate (Fig. 1C) of *L. plantarum*. Optical density in OC cultures increased 2.87-fold faster than under anode polarization (*P* = 0.019, two-tailed *t*-test).

Fermentation products showed a higher acetate fraction under anodic conditions in both acetate: lactate ratio and acetate yield per glucose, consistent with a more energetically favorable metabolism (Fig. 1B through D). By 38 h, OC cultures produced 36.71 ± 2.02 mM lactate and 0.56 ± 0.02 mM acetate, whereas anodic cultures produced 9.31 ± 0.34 mM and 0.66 ± 0.04 mM, representing a more than fourfold increase in acetate: lactate production (*P* < 0.0001 and *P* = 0.019 for lactate and acetate, respectively, two-tailed *t*-test). Since routing pyruvate to lactate, unlike acetate, regenerates NAD^+^, we considered whether the charge passed to the anode (Fig. 1E) was sufficient to allow the production of the observed acetate. By 38 h, an average of 181.2 coulombs of charge from the 300 mL medium volume had passed the anode corresponding to 6.26 mM electron equivalent, or the equivalent of NAD^+^ regeneration associated with making 3.13 mM lactate rather than acetate from pyruvate. On this basis, the current observed was more than sufficient to allow redox balancing for the increased acetate yield.

### The anode delays *L. plantarum* growth and fermentation on mannitol when acetate is available

**Fig 2:**
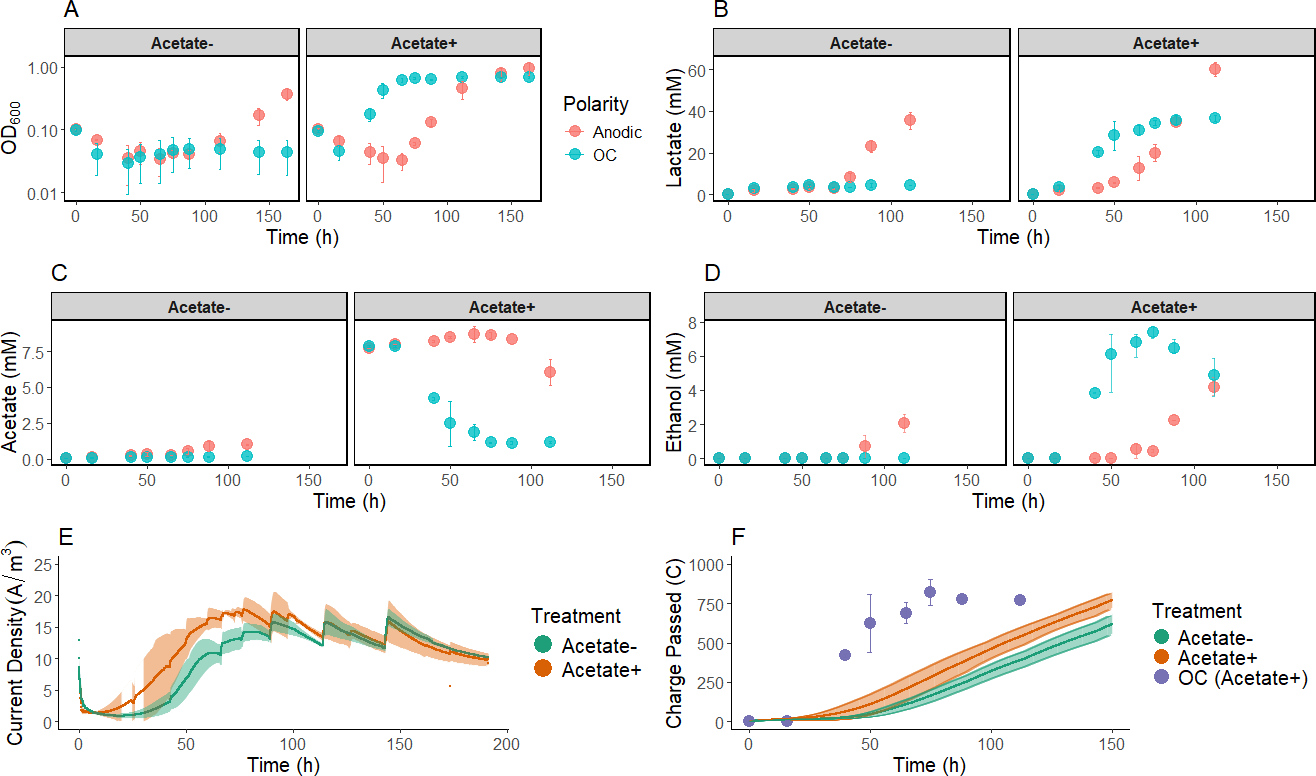
Batch fermentation of mannitol CDM with and without addition of acetate. (**A**) OD_600_, (**B**) lactate, (**C**) acetate, (**D**) ethanol concentrations, (**E**) chronoamperometric response, and (**F**) comparison of the cumulative charge (coulombs) passed to the anode with the charge equivalent associated with acetate-to-ethanol reduction in the OC condition (Appendix SA). Bars indicate the average ± standard deviation of biological triplicates. Metabolites were measured for the initial 112 h, capturing the consumption of acetate and exit of exponential phase in the control condition.

In comparison to glucose, mannitol is a more reduced substrate (average C oxidation state of −0.33 vs 0), necessitating different fermentation strategies to achieve redox balance. Anode polarization was previously associated with increased fermentation of mannitol in the same CDM (17). We next examined how an anode affects *L. plantarum* when another electron acceptor, acetate, is present in the media. In this context, acetate acts as a substrate for reduction to ethanol, an overlapping role with the anode as an electron acceptor. In the absence of both acetate and anode polarization, *L. plantarum* showed no evident growth as expected (Fig. 2A).

When comparing the effects of anode polarization on batch mannitol fermentation in the presence of acetate, anode polarization resulted in a markedly longer lag phase (ca. 75 h) than OC (ca. 26 h) (Fig. 2A). By 65 h, OD_600_ reached 0.62 ± 0.05 in OC cultures vs 0.03 ± 0.01 under anodic conditions (*n* = 3). Despite this delay, exponential growth rates were not significantly different (*P* = 0.47, two-tailed *t*-test). Anode polarization allowed continued growth to a higher final OD_600_ (0.95 ± 0.21), whereas OC cultures plateaued at 0.66 ± 0.04 from 65 to 164 h. Lactate accumulated as the principal metabolite in both conditions (Fig. 2B). An increase in ethanol (Fig. 2D) was observed concomitantly with acetate consumption in the OC condition, consistent with acetate-to-ethanol reduction as a route for NAD^+^ regeneration.

Despite the overlapping roles of acetate and the anode as electron acceptors for *L. plantarum*, a faster onset of current was observed with the presence of acetate than without acetate. Chronoamperometric responses converged after approximately 100 h of operation (Fig. 2E) despite higher cell density in the acetate+ group (Fig. 2A). Notable spikes of increased current are apparent immediately after pH adjustment (Fig. 2E), which was conducted concurrently with sampling.

Charge dissipation to acetate reduction occurred more rapidly than to the anode (Fig. 2F). At 75 h, 819.3 ± 83.2 coulombs had been dissipated to acetate in the OC group vs 161.1 ± 38.9 and 278.6 ± 76.5 to the anode in the groups with and without acetate, respectively, as determined by comparing the charge equivalents associated with the consumption of acetate (4 mol e^−^ per mol acetate to ethanol) with the integrated chronoamperometric curve. The anode blocked acetate consumption initially, with acetate increasing to 8.64 ± 0.20 mM by 75 h, followed by its consumption after the population entered the exponential phase (Fig. 2C).

To place these observations in the context of substrate redox state, glucose fermentation was examined as a less reduced substrate. Acetate addition to the CDM did not affect the glucose fermentation (Fig. S3). Neither acetate consumption nor the formation of detectable quantities of ethanol was observed. OCPs on glucose were similar with and without acetate, reaching −312 ± 2.43 mV vs Ag/AgCl after 38 h (Fig. 1F; Fig. S3F). The extracellular conditions were less reduced than when grown on mannitol, where OCPs reached minima of −476 ± 7 and −405 ± 4 mV vs Ag/AgCl in acetate+ and acetate− groups, respectively, after 53 h.

The relatively low concentration of riboflavin (1 mg L^−1^) supplied in these experiments may have limited the rate of EET, so experiments were repeated in an extreme example in the same mCDM with presence of acetate with riboflavin saturation. In this case, anodic and OC conditions did not have significantly different lag phases with similar OD_600_ after 64 h (*P* = 0.31), but an altered product distribution was observed (Fig. S4). More lactic acid was produced in the anodic group (45.20 ± 4.44 mM, *n* = 6) than the OC (32.19 ± 2.00 mM, *n* = 3) at 64 h, and on average, 4.01 mM less acetate was consumed (5.63 ± 1.05 mM, *n* = 6 remaining in anodic vs 1.62 ± 0.32 mM, *n* = 3 remaining in OC), and 5.89 mM less ethanol was produced (4.00 ± 0.78 mM, *n* = 6 in anodic, 9.89 ± 1.99 mM, *n* = 3 in OC).

### Anodic regulation leads to less growth in semi-continuous culture on mannitol

In a batch fermentation on mannitol without acetate, the anode serves as a necessary electron sink to regenerate NAD^+^. To assess its role under cultivation regimes with sustained metabolism, semi-continuous cultures were employed in which 25%, 50%, or 75% of the medium was replaced every 24 h for 6 days. This enables examination of growth and metabolism balance in a quasi-steady-state regime. In these experiments, the headspace composition was not controlled, and the pH was not adjusted.

**Fig 3:**
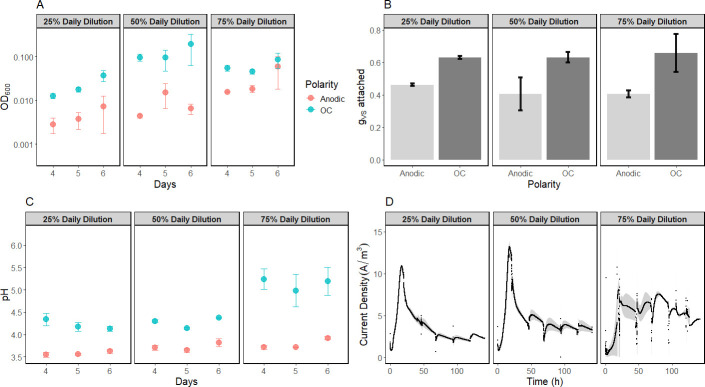
*L. plantarum* semi-continuous culture on mCDM grows less with anodic regulation. (**A**) OD_600_, (**B**) the mass of volatile solids attached to the carbon felt at the end of the experimental period, (**C**) pH, and (**D**) the chronoamperometric response. Bars indicate the range of duplicates.

Anode polarization resulted in lower planktonic and attached biomass (Fig. 3A and B). Anodic reactors had lower pH than OC, ranging from around 0.5 pH units lower at the slowest medium replacement rate to around 1.5 pH units lower at the 75% daily medium replacement rate (Fig. 3C). Biomass levels were lower in anodic reactors, likely reflecting the influences of acidic stress. Acetate concentrations averaged 1.82, 1.01, and 0.73 mM in the anodic groups at 25, 50, and 75% dilutions, respectively, compared to 0.25, 0.21, and 0.06 mM in OC groups.

Chronoamperometric responses increased with higher daily dilution (Fig. 3D). In batch culture without acetate or an anode as an electron sink, *L. plantarum* ended fermentation with minimal acidification (Fig. 2A), whereas semi-continuous cultivation supported continued growth and fermentation. This is attributable to the periodic introduction of dissolved oxygen with fresh media, allowing the facultative anaerobe *L. plantarum* to use oxygen as an alternative terminal electron acceptor. There was not a clear monotonic pattern between medium replacement rate and growth (Fig. 3A).

### Anode polarization and substrate affect gene expression

To understand the mechanism by which the anode impacted *L. plantarum* growth, RNA-seq was performed to compare the transcriptomes in early exponential phase batch culture between (i) anodic vs OC cultures grown on mCDM (mannitol, acetate+, saturation riboflavin) and (ii) anodic batch cultures grown on mannitol vs glucose (acetate+, saturation riboflavin), a total of three conditions (Fig. S4). Anode polarization significantly upregulated 112 genes during growth on mannitol versus the OC (Table S1, comparison 1), while 592 genes were differentially expressed between anodic fermentation of mannitol vs. glucose (Table S2, comparison 2).

The gene *pplA*, encoding a flavin-binding, extracellular surface-associated protein linked to flavin-based EET, was not differentially expressed in either comparison, nor was demethylmenaquinone methyltransferase, responsible for the conversion of DHNA to demethylmenaquinone, the putative link for electrons through the intramembrane space (16, 27).

Oxidative stress response genes reported to be induced by the addition of this same DHNA concentration in a rich media (mMRS) like those encoding thioredoxins, reductases, or thioredoxin family proteins, methionine-S-oxide reductase, or glutathione peroxidase (23) were not upregulated in anodic growth. Universal stress proteins were the first and fourth most strongly downregulated genes in the OC condition (comparison 1).

Growth on mannitol vs glucose (comparison 2) highly differentially expressed phosphotransferase system (PTS) components specific for reduced sugars (Fig. S5B). Major facilitator superfamily (MFS) transporters were upregulated on mannitol and may be responsible for acetate assimilation. Four alcohol dehydrogenase genes were also upregulated on mannitol, including, in order of increasing mean normalized expression in the mannitol condition, a NAD(P)-dependent alcohol dehydrogenase, an iron-containing alcohol dehydrogenase, and a bifunctional acetaldehyde/alcohol dehydrogenase (*adhE*). Upregulation of these genes relating to acetate transport and reduction was consistent with the observation of net acetate consumption and ethanol production during growth on mannitol, a process not observed on glucose.

### Prophage induction observed with anode polarization

**Fig 4:**
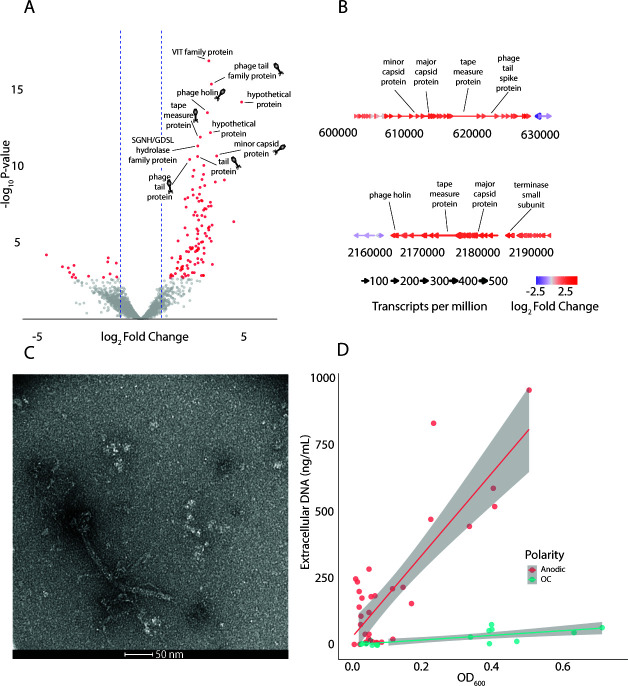
Anode polarization induces prophages in *L. plantarum*. (**A**) Comparison of DEGs from anodic vs OC mannitol fermentation (Comparison 1, mCDM, acetate+, saturation riboflavin) with top 10 DEGs by significance labeled. Among this group, prophage-annotated genes are labeled with a phage cartoon. Color indicates false discovery rate-adjusted *P* < 0.05 and an absolute value log_2_ fold change >1.0. (**B**) Details of the two prophage regions upregulated from the same conditions as panel A. Genes are labeled by average normalized abundance (transcripts per million [TPM]) across anodic and OC samples and by the log₂ fold change (anodic/OC). (**C**) TEM micrograph of negatively stained phage particle observed from batch culture in gCDM (acetate−, riboflavin only from Wolfe’s vitamins) in an anodically poised reactor. (**D**) Anode polarization leads to increased extracellular DNA during the lag and growth phase on glucose. Lines represent linear model fits with 95% confidence intervals (*n* = 4 anodic, *n* = 3 OC reactors).

Six of the top 10 DEGs upregulated by the anode on mannitol were assigned to prophage elements (Fig. 4A). PHASTEST analysis predicted two intact prophage regions in *L. plantarum* NCIMB8826 (LP_RS02850–LP_RS02935 and LP_RS10245–LP_RS10375), of which all genes were upregulated (Fig. 4B). Gene set enrichment analysis indicated that both prophage regions, LP_RS02850–LP_RS02935 (NES = 2.66, *P*_adj_ = 5.5 × 10^−^²⁰) and LP_RS10245–LP_RS10375 (NES = 2.97, *P*_adj_ = 4.8 × 10^−^²⁹), were significantly upregulated in anodically regulated compared with OC fermentations on mannitol. Thirty of the 112 upregulated DEGs were hypothetical proteins (Table S1), 22 of which were inside or adjacent to the two intact prophage regions. Prophage elements were similarly expressed during anodic growth on mannitol and glucose (Table S2).

TEM imaging revealed the presence of intact phage particles in exponential growth on batch cultures using gCDM with riboflavin only from Wolfe’s minerals (Fig. 4C), and in the riboflavin-saturated media used in the transcriptomic experiment (Fig. S6). The phages exhibited siphovirus-like morphology like those induced by mitomycin C and hydrogen peroxide from *L. plantarum* kimchi isolates (29). The prophage sequences in the reference strain WCFS1 were identified in 2003 but determined to be uninducible by mitomycin C and hydrogen peroxide (30).

Anode polarization increased the extracellular DNA, a proxy measurement of cell lysis, during growth on glucose (Fig. 4D). The relationship between OD_600_ and extracellular DNA during the lag and growth phase was strongly linear, determined by a linear mixed-effects model with the biological replicate as a random effect. DNA increased by 1,540 ng mL^−1^ per OD_600_ unit (*P* < 0.001) in the anodic condition, roughly 17-fold higher than in the OC. This amount of lysis could correspond to lysis of roughly 35% of the population—an estimate with caveats is provided in Appendix SB.

## DISCUSSION

### Charge transfer to acetate occurs more quickly than to the anode on mannitol

**Fig 5:**
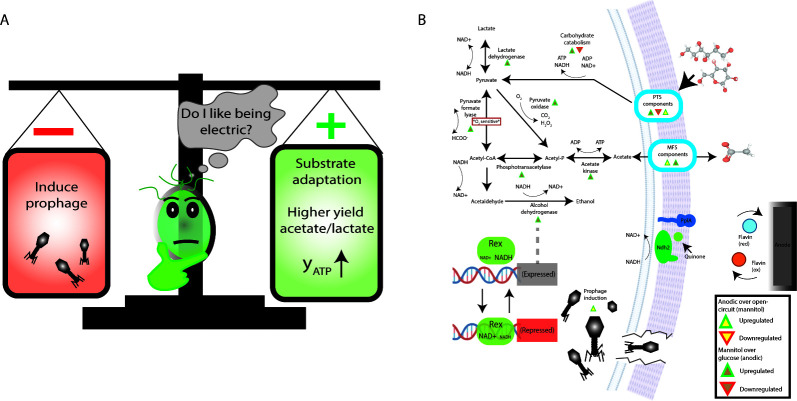
(**A**) A polarized anode can negatively and positively affect *L. plantarum*. (**B**) Summary of highlighted processes with differentially expressed genes indicated. Significant regulation indicated here as false discovery rate-adjusted *P* < 0.05 and absolute value log_2_ fold change > 1.0. This figure was created using elements from Biorender.com.

In batch culture on mannitol with acetate, a polarized anode as an alternate electron sink led to delay in growth (Fig. 2A), acetate assimilation/reduction (Fig. 2C), and slower charge dissipation (Fig. 2F) as compared with OC conditions. This was unexpected, as acetate reduction incurs an ATP cost associated with acetate activation by acetate kinase (Fig. 5). Acetate reduction depends on the bifunctional acetaldehyde/alcohol dehydrogenase, AdhE, which is regulated by the redox-sensitive, NADH-binding transcriptional repressor, Rex, in *L. plantarum* (9). The ability for *L. plantarum* to engage in outward EET during mannitol fermentation allows a more oxidized intracellular redox balance, reported as a NAD^+^/NADH ratio of ca. 50 at the time of maximum current, which is eightfold higher than the NAD^+^/NADH ratio in the OC (17). Anode polarization may increase intracellular redox potential sufficient to repress *adhE* via Rex (Fig. 5). Indeed, acetate was retained in anodic growth on mannitol until OD near 0.4 (Fig. 2C). Beyond this point, the rate of EET may have been insufficient to maintain a high enough NAD^+^/NADH ratio to hold Rex on, and a notable shift toward acetate consumption and ethanol production was observed. Although the chronoamperometric responses were similar during the later stages of this experiment (Fig. 2E), cell densities were much higher in acetate-amended cultures (Fig. 2A), suggesting a lower rate of EET per cell. From this alone, it is unclear why NAD^+^ regeneration through a pathway that is not expected to expressly cost ATP (FLEET) results in lower net growth than acetate reduction, which consumes one ATP by acetate activation per two NAD^+^ regenerated (Fig. 5).

### *adhE* regulation does not explain growth impairment on glucose

The anode was observed to retard growth (Fig. 1A) and fermentation (Fig. 1B) on glucose, even though EET is not required to achieve redox balancing. Transcriptomic and metabolite analyses indicate that acetate assimilation and reduction are not major routes for NAD^+^ regeneration (Fig. S3D and S5B), ruling out competition between soluble and electrode-based electron sinks as the cause of growth inhibition, as discussed above for mannitol. Instead, anodic electron transfer increased acetate yields, a shift that should increase ATP yield per substrate. This represents a unique scenario in which outward EET slows the net observed growth and substrate utilization despite promoting a more energetically favorable metabolic pathway.

### Implications of prophage expression

Prophage induction is suggested by this work to be a major factor influencing the physiology of *L. plantarum* during hybrid EET-fermentation involving an anode. The anode improved growth of *L*. plantarum only in the case of batch culture on mannitol without other electron acceptors (Fig. 2). In other environments, the anode decreased growth, as seen in semi-continuous culture on mannitol (Fig. 3) and batch culture on glucose (Fig. 1), or delayed growth, as in the case of batch culture on mannitol with acetate available (Fig. 2). Based on extracellular DNA measurements as a cell lysis proxy, a large fraction of the population, although less than 50%, is estimated to undergo lysis (Appendix SB). Prophage induction could reduce the net population growth rate measured by OD_600_, even if individual cells grow faster. Prophage deletion mutants and single-cell approaches (42) may help further elucidate the relationship between EET, prophage induction, and population-level growth outcome.

The mechanism driving prophage induction, whether EET itself or exposure to a polarized anode, cannot be concluded. For instance, quinone cycling could contribute to prophage induction. Partial quinone reduction (1e^-^) or quinol oxidation can form a semiquinone radical that reacts with oxygen to generate reactive oxygen species. In contrast, a prior study showed that supplying a soluble terminal electron acceptor (ferric ammonium citrate) alleviates DHNA-associated growth impairment and transcriptional responses associated with oxidative stress in the same strain of *L. plantarum* (23), but prophage expression was not reported. Comparing our results with this report suggests that the physiological effects of quinones and/or EET depend on the nature of the terminal electron acceptor (i.e., soluble/insoluble iron vs. anode). Further work should involve EET-deficient mutants. Furthermore, in environmental systems, oxygen-scavenging heterotrophs may mitigate harm caused by quinone redox cycling by limiting reactive oxygen species formation.

The association between polarization of the anode with upregulation of prophage transcripts, production of intact virions, and higher lysis rates suggests a possible link between the action of the prophage and the growth impediment, which deserves future experiments on prophage knockouts in this strain and screening the electrode’s effects on other strains. *L. plantarum*’s EET capability (17) and prophage induction mechanisms/capacity (28–30) vary among strains, adding nuance to the application of EET and electrodes for fermentation control. The role of polylysogeny should also be further explored: the two prophages upregulated here share 88% DNA sequence identity over about 10 kb but differ in some key regions, including integration sites (30).

From an ecological perspective, the link between EET and prophage induction has implications for microbial interactions. For example, the released phage particles may target competing cells (43). Since *L. plantarum* relies on mediators as “public goods” from neighbor cells, it is possible that mediator secretion contributes to prophage induction in *L. plantarum*. In addition, lysate mediated by phage may release nutrient and promote the growth of neighbor cells (44).

Further, mobile genetic elements have been suggested to benefit the host organism by encoding redox balancing functionalities, like prophages in *Geobacter soli* (45) and borgs associated with *Methanoperedens* spp. (46). In contrasting fashion, prophage expression here is implicated with slower population growth, and genes known to be central to redox balancing are not found inside or adjacent to prophage regions. The connection between electroactivity and prophage induction could be further contextualized to more taxa and different environmental conditions.

### Conclusion

EET to an anode, which decouples central carbon flow from redox balancing requirements, is shown to have a variable physiological effect on *L. plantarum*, conferring a boost in the absence of other redox-balancing strategies, like during growth on mannitol, but slowing or delaying growth in other conditions where it is not needed to balance charge. We describe here how an anode can promote more favorable fermentation patterns, coincident with impediment of population-level growth and prophage induction—a novel connection between redox balancing and mobile genetic elements in fermentation. The polarization of an anode is associated with induction of intact prophages, which have not been reported from this strain. More practically, these findings advance understanding of how anodically-poised electrodes can be used to regulate fermentation of reduced substrates. Finally, this connection between electroactivity and prophage induction raises the question of how electroactive fermenters without intact prophages may be controlled by the electrode differently.

## Data Availability

RNA-seq fastq files are available on the NCBI SRA (BioProject PRJNA1196378).

## SUPPLEMENTAL MATERIAL

Supplemental Material msystems.00183-26-s0001.docx Figures S1 to S6, Appendix SA, and Appendix SB.

Table S1 msystems.00183-26-s0002.xlsx DEGs, anodic vs open circuit (mannitol).

Table S2 msystems.00183-26-s0003.xlsx DEGs, mannitol vs glucose.

ASM does not own the copyrights to Supplemental Material that may be linked to, or accessed through, an article. The authors have granted ASM a non-exclusive, world-wide license to publish the Supplemental Material files. Please contact the corresponding author directly for reuse.
