# Supplementary material for: Prophage induction and acetate availability are associated with distinct Lactiplantibacillus plantarum electrode responses
Source: mSystems. 2026 Apr 30;11(5):e00183-26. doi: 10.1128/msystems.00183-26 (PMC13185627; doi:10.1128/msystems.00183-26)
Supplement: Supplemental Material — Figures S1 to S6, Appendix SA, and Appendix SB. [file msystems.00183-26-s0001.docx]

**Supplementary information and appendices**


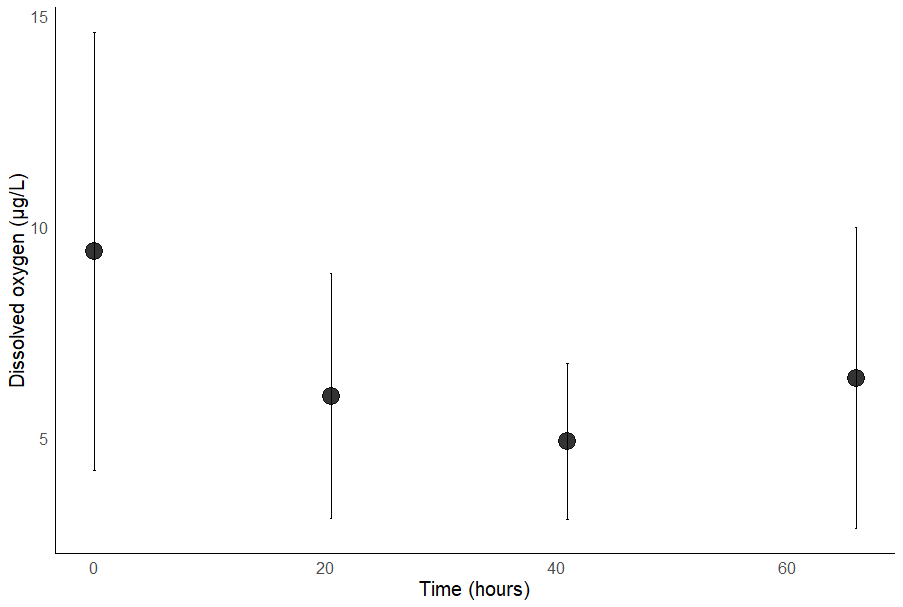


**Figure S1** – Dissolved oxygen profile in the working electrode chamber over the course of *L. plantarum* growth as measured by PreSens optical sensor spot (SP-PSt3-YAU, Regensburg, Germany) (n=4, average ± standard deviation).


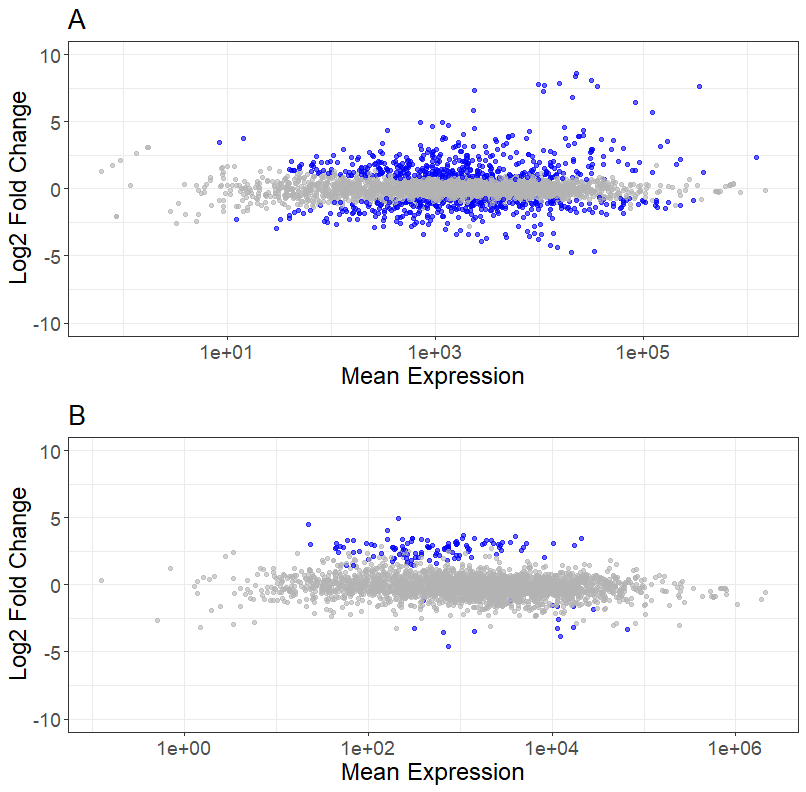


**Figure S2** – MA plots of DESeq2 outputs for mannitol/glucose (A) and anodic/OC (B) comparisons. DEGs (false discovery rate-adjusted *p* < 0.05 are shown in blue).


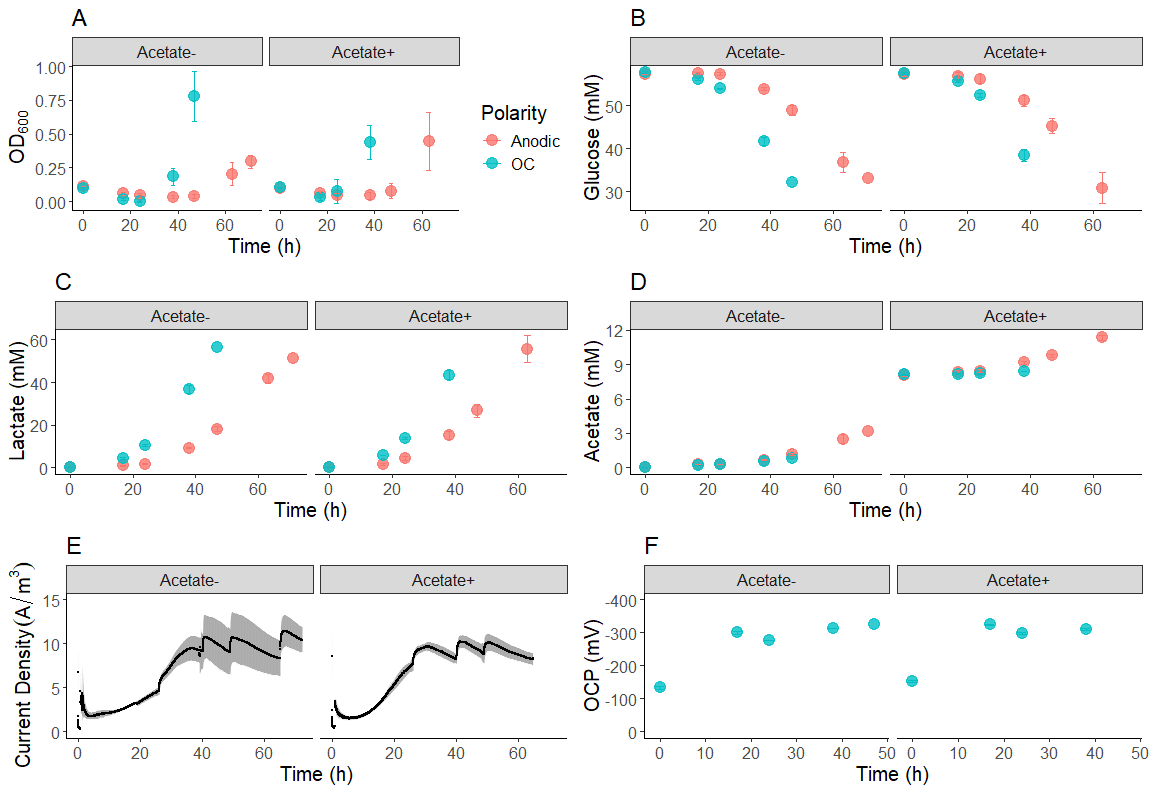


**Figure S3 -** Anodic polarization impedes *L. plantarum* glucose fermentation batch cultures with and without addition of acetate. Fermentations conducted in gCDM until OD_600_ ≥0.2. (A) OD_600_, (B) glucose, (C) lactate, (D) acetate, (E) chronoamperometric response, and (F) OCPs were measured from biological triplicates and plotted. Bars indicate the average ± standard deviation of triplicates.


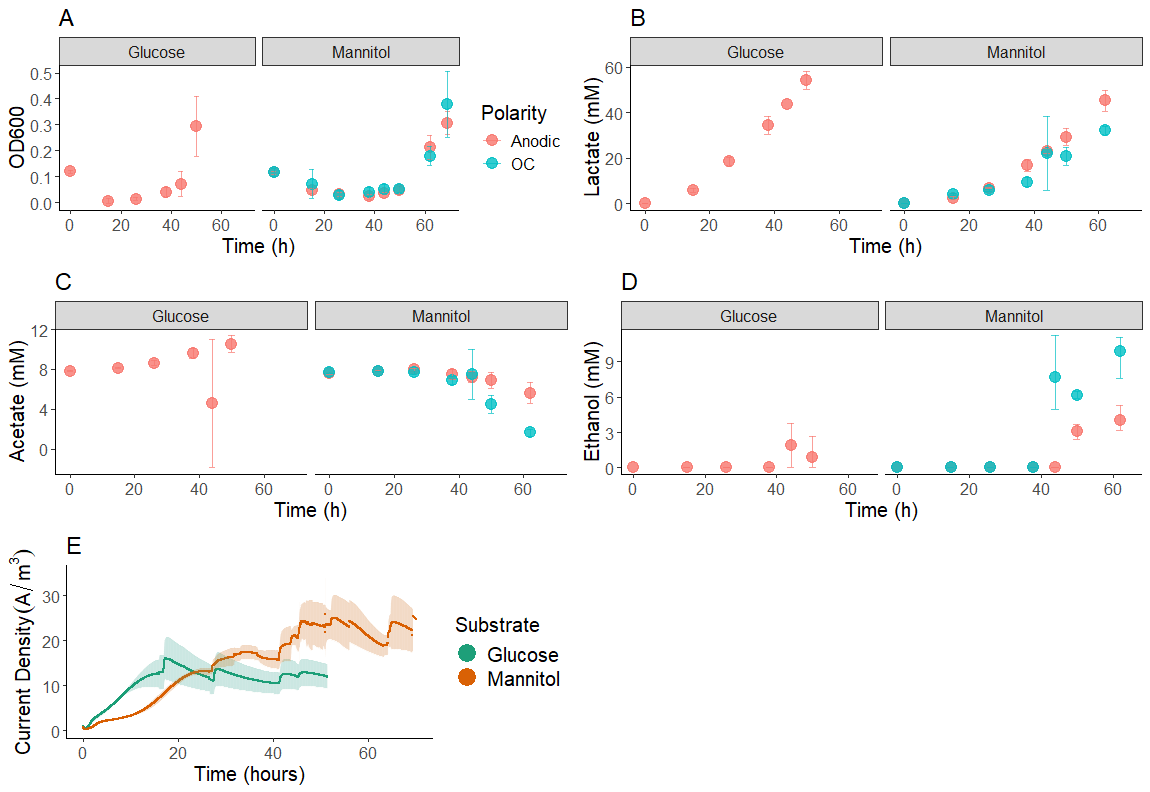


**Figure S4** – Data from RNA-seq experiment. Comparison of OC (n=3) vs. anodic (n=6) mannitol fermentation and anodic (n=3) glucose fermentation. Experiments conducted in g/mCDM with saturation riboflavin. Extracted RNA from three anodic/mannitol reactors had unsatisfactory RIN values (< 7.0) and were excluded from the RNA sequencing analysis.


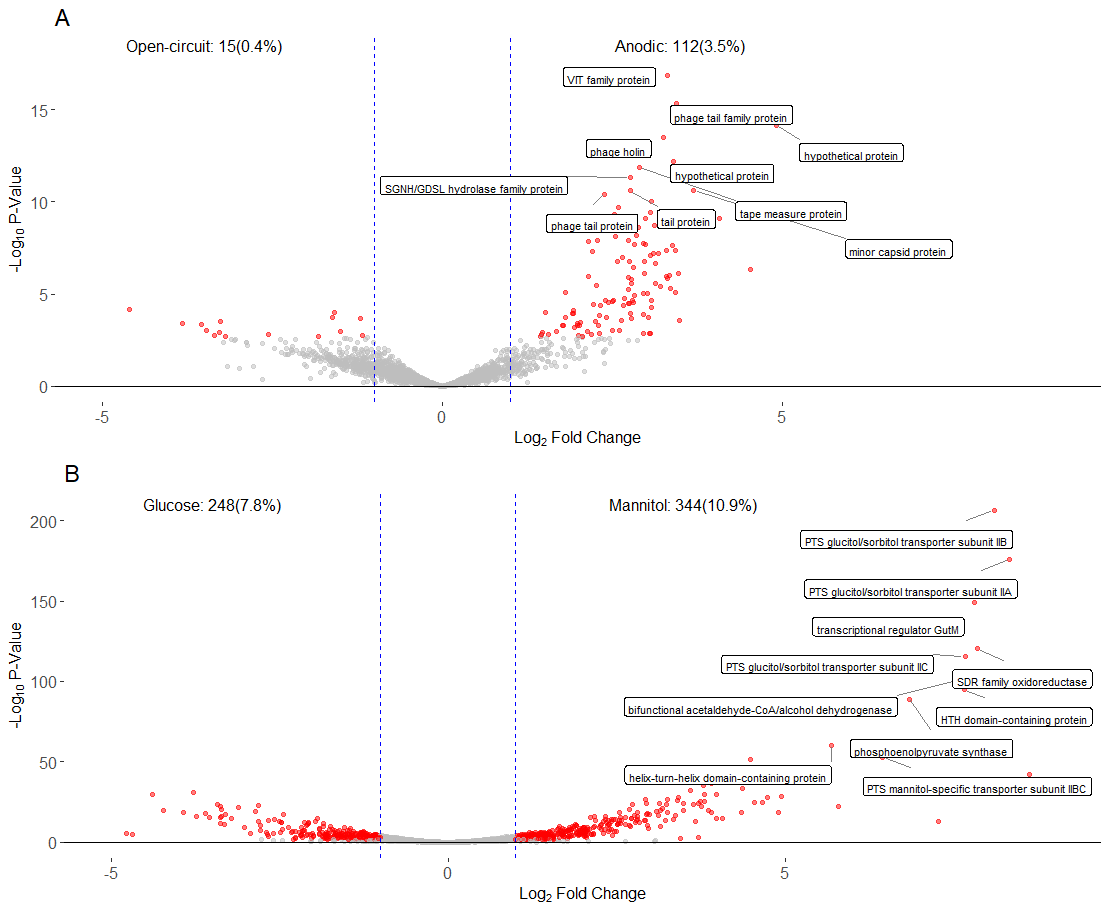


**Figure S5** – Volcano plots showing DEGs from (A) anodic vs. OC mannitol fermentation and (B) mannitol vs. glucose fermentation under anodic regulation (Acetate+). Top 10 genes by significance are indicated. Color indicates false discovery rate-adjusted p-value <0.05 and an absolute value log_2_ fold change >1.0.


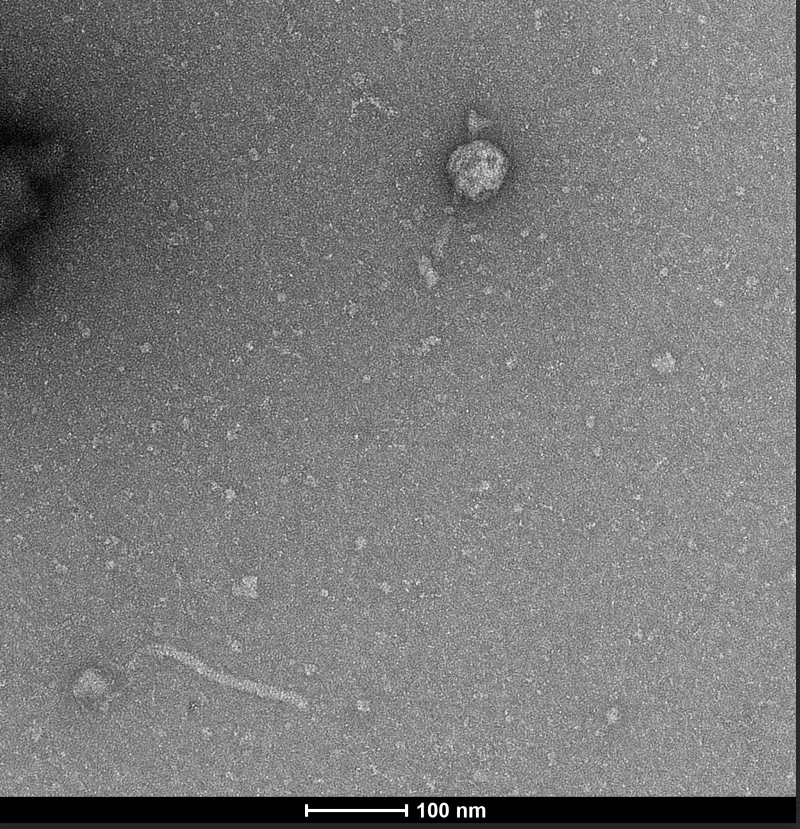


**Figure S6** -TEM micrograph of phage particles during exponential growth on gCDM (acetate+, saturation riboflavin)

**Supplemental Material Appendix A - Electrochemical analyses calculations**

An estimate of the NAD+ via extracellular electron transport vs. acetate reduction is quantitatively compared in Figure 2F. The charge passed to the anode via redox mediators and the charge passed to acetate are compared.

The charge (coulombs, Q) passed to the anode is found via integration of the chronoamperometric curve:


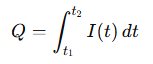


The charge associated with the reduction of acetate to ethanol follows the stoichiometry


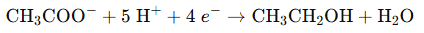


Therefore, the consumption of a mol acetate consumes 4 moles of electrons.

**Supplemental Material Appendix B - Contextualization of extracellular DNA vs. OD relationship**

It is meaningful to contextualize the approximate number of cells that lysed in order to yield the observed extracellular DNA in Figure 3C.

*L. plantarum* WCFS1genome size ≈ 3.3 Mbp (3.3 × 10⁶ bp)

1 bp ≈ 660 Da → 3.3×10⁶ × 660 Da ≈ 2.18×10⁹ Da per genome

1 Da = 1.66×10⁻²⁴ g → DNA mass per cell ≈ 2.18×10⁹ × 1.66×10⁻²⁴ g ≈ 3.62×10⁻¹⁵ g/cell ≈ 3.6 fg/cell

At an optical density of 0.4, an extracellular DNA concentration of roughly 500 ng/mL = 500 × 10⁻⁹ g/mL is observed in the anodic condition


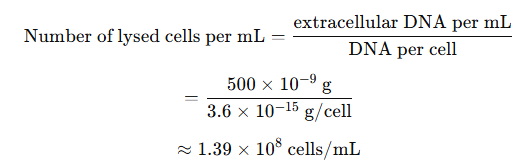


Assume roughly 0.4 * 10⁹ CFU/mL at an OD of 0.4 (Trabelsi *et al.* 2013)

Caveat - different *L. plantarum* strain in rich media

1.39/4 ~ 0.35

Since extracellular DNA appears to scale linearly with growth, a reasonable estimate is that 35% of cells have lysed

Caveat - extracellular DNA may be of non-lytic origin

Caveat – Lysed cells are expected to have some contribution to OD

Trabelsi, I., Bejar, W., Ayadi, D., Chouayekh, H., Kammoun, R., Bejar, S. and Salah, R.B., 2013. Encapsulation in alginate and alginate coated-chitosan improved the survival of newly probiotic in oxgall and gastric juice. *International journal of biological macromolecules*, *61*, pp.36-42.
